# Endocannabinoids Have Opposing Effects On Behavioral Responses To Nociceptive And Non-nociceptive Stimuli

**DOI:** 10.1038/s41598-017-06114-1

**Published:** 2017-07-19

**Authors:** Torrie Summers, Brandon Hanten, Warren Peterson, Brian Burrell

**Affiliations:** 1 Division of Basic Biomedical Sciences, Center for Brain and Behavior Research, Sanford School of Medicine, University of South Dakota, Vermillion, SD 57069 USA; 2 Riot Games, Santa Monica, CA USA

## Abstract

The endocannabinoid system is thought to modulate nociceptive signaling making it a potential therapeutic target for treating pain. However, there is evidence that endocannabinoids have both pro- and anti-nociceptive effects. In previous studies using *Hirudo verbana* (the medicinal leech), endocannabinoids were found to depress nociceptive synapses, but enhance non-nociceptive synapses. Here we examined whether endocannabinoids have similar bidirectional effects on behavioral responses to nociceptive vs. non-nociceptive stimuli *in vivo*. *Hirudo* were injected with either the 2-arachidonoylglycerol (2-AG) or anandamide and tested for changes in response to nociceptive and non-nociceptive stimuli. Both endocannabinoids enhanced responses to non-nociceptive stimuli and reduced responses to nociceptive stimuli. These pro- and anti-nociceptive effects were blocked by co-injection of a TRPV channel inhibitor, which are thought to function as an endocannabinoid receptor. In experiments to determine the effects of endocannabinoids on animals that had undergone injury-induced sensitization, 2-AG and anandamide diminished sensitization to nociceptive stimuli although the effects of 2-AG were longer lasting. Sensitized responses to non-nociceptive stimuli were unaffected 2-AG or anandamide. These results provide evidence that endocannabinoids can have opposing effects on nociceptive vs. non-nociceptive pathways and suggest that cannabinoid-based therapies may be more appropriate for treating pain disorders in which hyperalgesia and not allodynia is the primary symptom.

## Introduction

There is considerable interest in utilizing cannabinoid-based therapies to treat pain^1, 2^. Endogenous cannabinoid transmitters (endocannabinoids), such as 2-arachidonoyl (2-AG) and anandamide, have been shown to decrease nociceptive signaling at the level of the spinal cord or to alleviate pain tested at the behavioral level^3–7^. However, preclinical studies have found that endocannabinoids can also enhance nociception^8, 9^. These findings may explain why some clinical studies of cannabinoid-based analgesic therapies either failed to reduce or even increased chronic pain symptoms^10–12^. Understanding how endocannabinoids can have both pro- and anti-nociceptive effects would improve the therapeutic potential of cannabinoid-based treatments by elucidating what types of pain symptoms, i.e. hyperalgesia and allodynia, are appropriate to be treated using cannabinoid-based drugs.

At the physiological level the opposing effects on endocannabinoids on nociception are based, at least in part, on the ability of these transmitters to depress both excitatory (glutamatergic) and inhibitory (GABAergic or glycinergic) synapses^13^. Depression of excitatory central synapses would be expected to lead to a decrease in nociceptive circuit output and ultimately an analgesic effect^7^. Depression of inhibitory synapses, however, could lead to disinhibition of nociceptive circuits, producing an increase in circuit output and enhancing pain signaling^8^.

The major barrier in understanding the pro- and anti-nociceptive effects of endocannabinoids is linking the behavioral effects to specific elements of the nociceptive circuitry. An especially difficult issue involves examining the potential role of non-nociceptive afferents that have access to nociceptive microcircuits^14, 15^. This access is regulated by inhibitory neurons that effectively control or “gate” whether non-nociceptive afferents have input to nociceptive microcircuits^16–18^. Studies using *Hirudo verbana* (the medicinal leech) provide an approach that can help to overcome this barrier. The central nervous system (CNS) of *Hirudo* is arranged as a chain of ganglia with each ganglion having its own compliment of sensory, motor and interneurons^19^. Furthermore, the identity and function of many neurons in each of these ganglia is known in considerable detail^20^. In terms of somatosensory signaling, the *Hirudo* CNS possesses three bilateral pairs of rapidly-adapting touch-sensitive neurons (T cells), two pairs of slow-adapting pressure-sensitive neurons (P cells) and two pairs of high-threshold nociceptive neurons (N cells)^21^. The N cells can be further divided into mechanical and polymodal nociceptors, with the latter being sensitive to noxious mechanical, thermal and chemical stimuli, e.g., H^+^, capsaicin and mustard oil^22–25^. P cell stimulation is capable of producing localized withdrawals from mechanical stimuli referred to as local bending and local shortening^26–28^. *Hirudo* are also capable of a whole body shortening reflex in which the entire animal withdraws from a noxious stimulus in a coordinated manner^29^. Whole-body shortening can be elicited by the P cells if multiple P cell receptive fields are activated, whereas this reflex can be activated by a single N cell^29, 30^.

**Figure 1:**
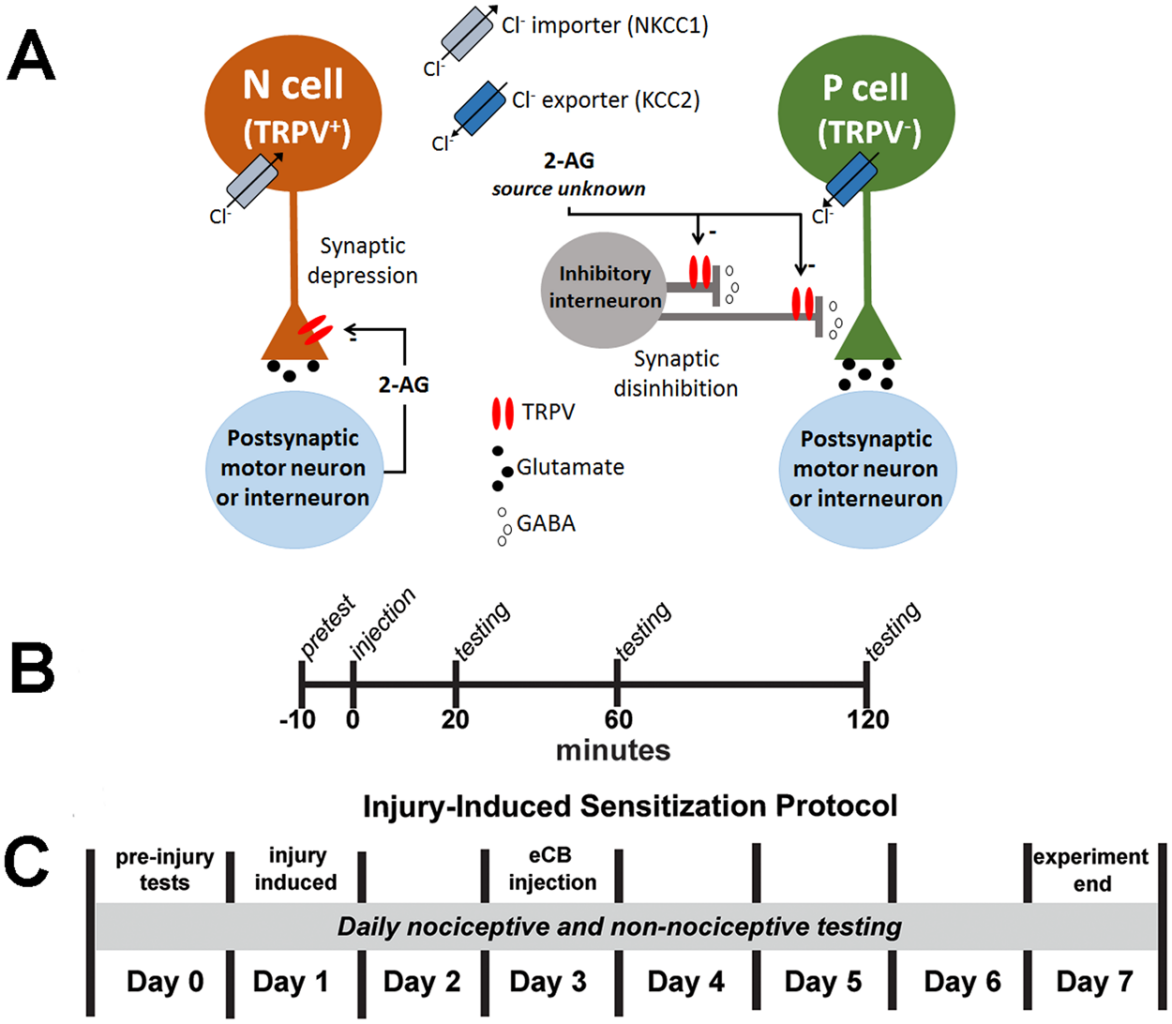
Endocannabinoid-mediated synaptic plasticity in Hirudo synapses and experimental protocols. (**A**) Endocannabinoids directly depress the nociceptive (N) synapse via a presynaptic TRPV-like receptor that reduces neurotransmitter release^31, 34, 35^. Endocannabinoids potentiate the non-nociceptive synapse (P) via an indirect mechanism in which endocannabinoids depress inhibitory input from an unknown GABAergic interneuron (this is also TRPV-mediated). In N cells the Cl^−^ gradient is dominated by the Cl^−^ importer (NKCC1) while the P cell gradient is dominated by the Cl^−^ exporter (KCC1)^36^. As a result of an elevated intracellular Cl^−^ concentration, N cells are depolarized by GABA and consequently “protected” from disinhibition. (**B**) For tests of endocannabinoid effects in uninjured animals, *Hirudo* were given a pre-test to assess initial responsiveness to non-nociceptive and nociceptive stimuli 10 minutes prior to injection of either anandamide (100 μM) or 2-AG (75 μM), with or without 25 μM SB366791, subsequent tests of nociceptive and non-nociceptive responses were given 20, 60, and 120 mins post-injection. (**C**) For tests of endocannabinoid effects in animals that had undergone injury-induced sensitization, *Hirudo* responses to non-nociceptive and nociceptive stimuli were first assessed on day 0. On day 1, animals received a crush injury and responses to non-nociceptive and nociceptive stimuli were tested daily. On day 3, some animals received injections of 2-AG or anandamide.

Previous studies in *Hirudo* have shown that 2-AG and anandamide elicit long-lasting (≥1 hr) depression in nociceptive N cell synapses and potentiation in non-nociceptive P cell synapses (summarized in Fig. 1A)^30–35^. These studies suggest that endocannabinoid effects on both synapses are mediated by a TRPV-like channel. The synaptic effects of 2-AG have also been observed at the behavioral level using semi-intact preparations in which it possible to monitor both physiological and behavioral changes. Specifically, N cell elicited whole-body shortening was reduced by 2-AG^30^. However, there has been no attempt to examine whether the effects observed in such reduce preparations can also be seen in intact animals. Therefore, the current study examined the effects of 2-AG and anandamide on behaviors elicited by non-nociceptive vs. nociceptive stimuli *in vivo*. Consistent with our earlier physiological studies, 2-AG and anandamide enhanced responses to non-nociceptive mechanical stimuli and reduced responses to nociceptive chemical stimuli. Furthermore, in animals that had undergone injury-induced sensitization, 2-AG and anandamide reversed the sensitized responses to nociceptive stimuli, but had no effect on sensitized responses to non-nociceptive stimuli.

## Methods

### Animals and behavioral protocols

Leeches (*Hirudo verbana*; *3* *g*) were obtained from commercial suppliers (Leeches USA, Westbury, NY or Niagara Leeches, Cheyenne, WY) and maintained in artificial pond water [0.52 g/L H_2_O Hirudo salt (Leeches USA Ltd.)] on a 12 hour light/dark cycle at 15 °C in a refrigerated incubator.

The techniques for testing *Hirudo* responses to nociceptive and non-nociceptive stimuli are based on previously published protocols^25, 36^. Individual *Hirudo* were placed in a testing arena consisting of a plastic petri dish (145 mm diameter) lined with filter paper that had been saturated with pond water and maintained at room temperature. All animals were allowed to acclimate to the testing arena for 30 minutes prior to the start of the experiments. In experiments not involving injury-induced sensitization, each animal was initially tested (pre-test) for responses to nociceptive and non-nociceptive stimuli, followed by drug injection and then a post-test measurement for changes in responses to these stimuli at 20, 60 and 120 minutes post-injection (Fig. 1B).

For tests of responses to non-nociceptive stimulus an ascending range of von Frey filaments (0.008–2.0 g) was applied to the posterior sucker at 30-second inter-trial intervals. The threshold for a behavioral response was defined as the first von Frey fiber to elicit a localized shortening response that did not involve the sucker being picked up and withdrawn from the site of stimulation, which would correspond to a whole-body shortening response^37^. The range of von Frey fibers used to elicit this localized shortening behavior are well below the level necessary to elicit responses from the N cells^21, 23, 38^.

For a nociceptive stimuli, 800 μL of 250 μM allyl isothiocyanate (AITC, the active component of mustard oil) was applied to the external surface of the posterior sucker using a pipette similar to what we have previously reported^25^. AITC has been shown to elicit nocifensive responses in invertebrates possessing a TRPA1 channel homolog^39, 40^. Previous studies in our lab have found that the *Hirudo* polymodal N cell does respond to peripheral application of AITC, which elicits withdrawal of the posterior sucker from the site of application^25^. The magnitude of this withdrawal reflex was noticeably greater than the responses elicited by the von Frey fibers and likely corresponds to a whole-body shortening response^29^. Responses to mustard oil were video recorded and subsequently analyzed using Noldus Observer XT software. Nocifensive behaviors were quantified in terms of latency to withdraw. The person performing these analyses was blind to the experimental conditions (i.e. drug treatment and/or injury status) of each animal. Animals were excluded from further analysis if during the pre-test they were observed to have begun to withdraw their sucker either immediately before or just as the AITC was applied to the animal (7 out of 109 animals tested). Following the nociceptive stimuli tests, all animals were rinsed off for 20 seconds with leech pond water and placed in a clean testing arena. The pre-injection behavioral measure (response threshold, response latency) were used to represent 100% for each animal tested. Consequently, the subsequent post-injection behavioral measure were normalized to the pre-injection level for each animal (subsequent statistical analyses only used the post-injection data).

### Endocannabinoid injections

Drugs used for each experiment were kept as frozen aliquot solutions and then diluted to their final concentration in normal *Hirudo* saline (110 mM NaCl, 5 mM NaOH, 4 mM KCl, 1.8 mM CaCl_2_, 1 mM MgCl_2_, and 10 mM HEPES, pH = 7.4) just before the start of the experiment. Allyl isothiocyanate (AITC), 2-arachidonylglycerol (2-AG), and anandamide stocks were made in dimethyl sulfoxide (DMSO). 2-AG, anandamide DMSO, and SB366791 (TRPV1 antagonist) were obtained from Tocris (Ellisville, MO), while AITC was obtained from Sigma-Aldrich (St. Louis, MO).

Prior to drug injections, animals were lightly anesthetized with ice-cold saline in an ice-lined dissecting dish and injected with 100 μL of either 100 μM anandamide, 100 μM of anandamide +25 μM of SB366791, 75 μM 2-AG, or 75 μM 2-AG +25 μM SB366791. Pilot studies examining the effects of SB366791 injections found that concentrations greater than 25 μM reduced responses to nociceptive stimuli. For vehicle control experiments, 100 μL of 0.01% DMSO were injected. As previously reported^36^, injections were made just anterior of the posterior sucker, a region where the dorsal and ventral sinuses that are part of the leech vascular system converge^19^. The leech CNS is contained within the ventral sinus so this method of injection is likely to be effective in delivering drugs to the CNS.

### Injury-induced sensitization

The experimental protocol is summarized in Fig. 1C. On Day 0 (D0) pre-injury thresholds to non-nociceptive stimuli (von Frey fibers) and responses to nociceptive stimuli (AITC application) were measured as described previously. Injury-induced sensitization was delivered on Day 1 (D1). Each animal was initially anesthetized with ice-cold saline in an ice-lined dish for 20 seconds. Next, the posterior sucker was crushed for 20 seconds using a 13 cm long hemostat (crush dimensions were approximately 9 mm by 2.5 mm). This approach was used to produce an ethologically-relevant form of injury-induced sensitization that mimicked a potential injury produced by a predators bite. No obvious changes in sucker motor function were observed following application of this crush injury. Responses to mechanical and chemical stimuli were assessed one hour after injury and then each day for seven days. On day 3 (D3) each animal received an injection 100 μL of either 0.01% DMSO (vehicle control), anandamide (100, 75, or 25 μM) or 2-AG (75, 50, or 25 μM). Control, non-injured animals also receive a DMSO injection.

### Statistical Analysis

Behavioral data were presented as mean ± standard error. Results were normalized to pre-test results for both nociceptive and non-nociceptive experiments. Statistical analyses using two-way analysis of variance (ANOVA) were performed to determine the main effects with Student-Newman-Keuls post-hoc to confirm the ANOVA results.

## Results

### Opposing effects of endocannabinoids are prevented by a TRPV inhibitor

First, the effects of endocannabinoid injections on *Hirudo* responses to mechanical non-nociceptive stimuli and chemical nociceptive stimuli were assessed. Mechanical non-nociceptive stimuli were delivered via von Frey fibers that apply force sufficient to activate the non-nociceptive sensory neurons in *Hirudo* (0.008 g–2.0 g), but were below the mechanical threshold for activating mechano-nociceptive cells (7.0 g)^21^. Chemical nociceptive stimuli were delivered via the application of 250 μM AITC (800 μL) to the posterior sucker as carried out in previous experiments with *Hirudo* ^25, 36^. The von Frey fibers elicit a localized shortening responses, whereas AITC elicits whole body shortening in which the animal picks up its sucker to withdraw it from the site of AITC application.

**Figure 2:**
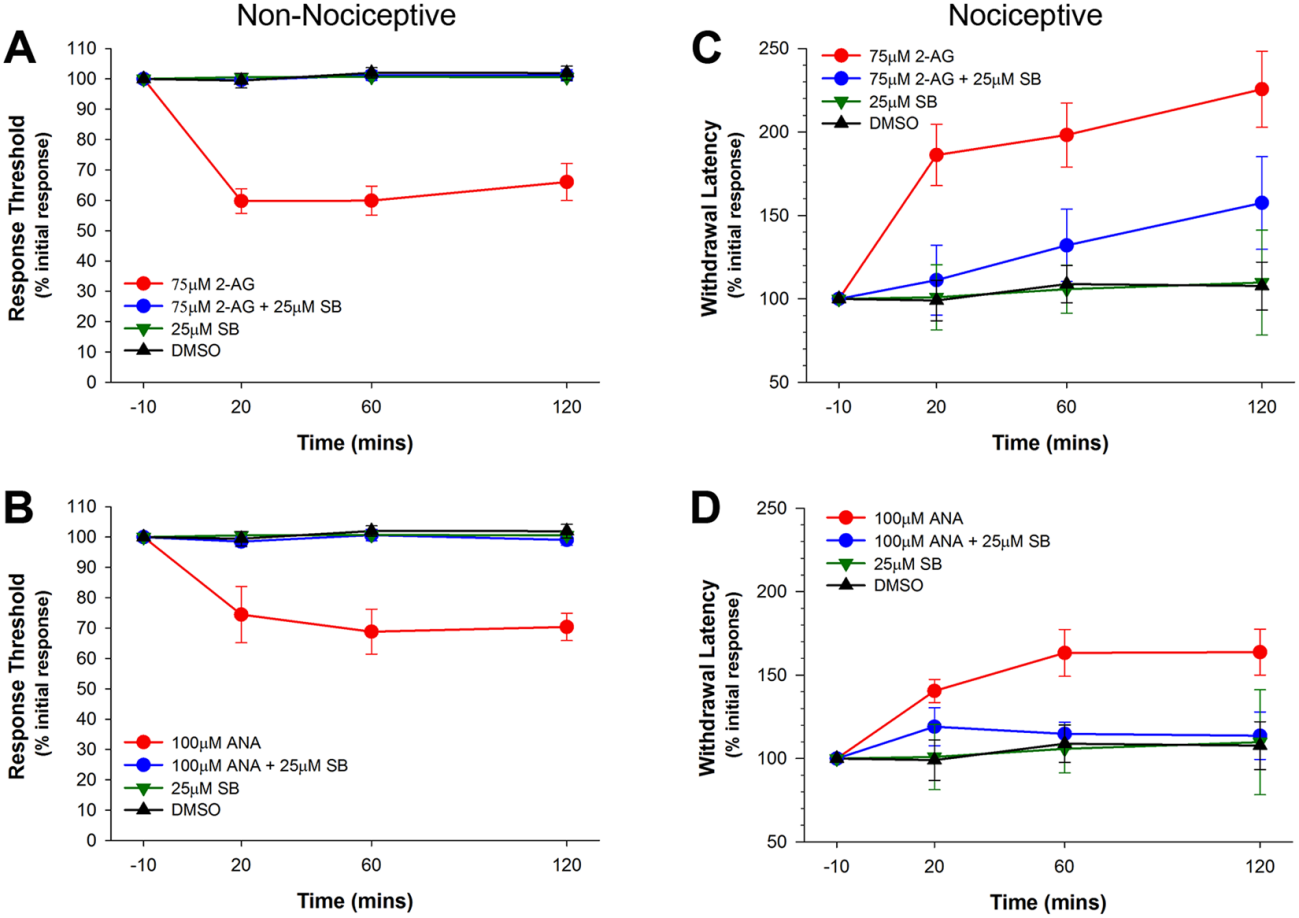
Differential effects of endocannabinoid treatment on responses to non-nociceptive and nociceptive stimuli in non-injured animals. (**A**) Injection of 2-AG reduced the response threshold to non-nociceptive mechanical stimuli and this effect was blocked by co-injection of the TRPV1 inhibitor SB366791 (SB). (**B**) Injection of anandamide (ANA) also reduced the response threshold to non-nociceptive mechanical stimuli and this was blocked with co-injection of SB366791. (**C**) 2-AG increased the latency to respond to nociceptive chemical stimuli (AITC) and this effect was blocked by SB366791. (**D**) Anandamide also increased the latency to respond to nociceptive chemical stimuli (AITC) and this effect was blocked by SB366791.

Following 2-AG (75 µM) injections the response threshold to non-nociceptive stimuli decreased relative to pre-test levels for the entire 120 minute testing period (Fig. 2A). Two-way ANOVA of the data collected during the three post-injection tests detected a statistically significant effect of drug treatment (F_3,98_ = 88.50, p < 0.001), but no significant effect of time (F_2,98_ = 0.10, p > 0.05) nor drug-time interaction effect (F_6,98_ = 0.43, p > 0.05). A post-hoc comparison confirmed that the normalize response threshold of 75 µM 2-AG group (N = 5) was significantly lower when compared to the DMSO control group (N = 12). When the TRPV1 antagonist SB366791 (25 μM) was co-injected with the 2-AG (N = 5), the 2-AG-elicited change in response threshold was no longer observed (Fig. 2A; p < 0.001 for 2-AG vs. 2-AG + SB366791 post-hoc test). SB366791 by itself (N = 5) had no effect on response threshold (p > 0.05).

Identical results were observed following anandamide injection (100 µM). Response thresholds were significantly reduced for the full 120 minute period following anandamide injection (Fig. 2B; F_3,83_ = 38.70, p < 0.001) with no significant effect of time (F_2,83_ = 0.24, p > 0.05) nor drug-time interaction (F_6,83_ = 0.25, p > 0.05). As with 2-AG, co-injection of SB366791 completely blocked anandamide’s effect (Fig. 2B; p < 0.001 for anandamide vs. anandamide + SB366791 post-hoc test). A post-hoc comparison confirmed that the normalize response threshold of 100 µM anandamide group (N = 6) was significantly lower when compared to the DMSO control group (N = 11; P < 0.001). When the SB366791 (25 μM) was co-injected with the anandamide (N = 6), the anandamide-elicited change in response threshold was no longer observed (Fig. 2B; p < 0.001 for anandamide vs. anandamide + SB366791 post-hoc test). Once again SB366791 by itself (N = 5) had no effect on response threshold (p > 0.05).

Next, the responses to a noxious chemical stimulus (AITC) topically applied to the posterior sucker were tested in endocannabinoid-treated animals. 2-AG-injection produced an increase in the response latency to noxious chemical stimuli over the 120 min testing period (Fig. 2C). Two-way ANOVA detected a statistically significant effect of drug treatment on withdrawal latency (F_3,86_ = 19.09, p < 0.001), but no significant effect of time (F_2,86_ = 1.71, p > 0.05) nor drug-time interaction (F_6,86_ = 0.30, p > 0.05). A post-hoc comparison confirmed that the normalize withdrawal latencies of the 75 µM 2-AG group was significantly higher when compared to the DMSO control group (sample sized are identical to those for the non-nociceptive tests). Co-injection of SB366791 significantly attenuated the effect of both 2-AG on response latency (Fig. 2C; p < 0.001 for 2-AG vs. 2-AG + SB366791 comparison). Although there appears to be an increase in latency in the 2-AG + SB366791 group at the 120 min post-injection, no statistically significant different was observed between the 2-AG + SB366791 group and the DMSO control group. No change in response latency was observed between in the *Hirudo* injected with SB366791 by itself (Fig. 2C; p ≥ 0.05).

Anandamide also produced a statistically significant increase in response latency to AITC application (Fig. 2D). Two-way ANOVA detected significant treatment effect (F_3,92_ = 8.91, p < 0.001) with no significant effect of time following injection (F_2,92_ = 0.64, p > 0.05) nor drug-time interaction (F_6,92_ = 0.13, p > 0.05). Subsequent post-hoc analysis did confirm significant difference between the anandamide- and DMSO-injected groups (p < 0.001). As with 2-AG, co-injection of SB366791 significantly attenuated the effect of anandamide on response latency (Fig. 2D; p < 0.001). Animals treated with SB366791 alone exhibited no significant changes in withdrawal latency (p ≥ 0.05).

### Effect of endocannabinoids on injury-induced sensitization

**Figure 3:**
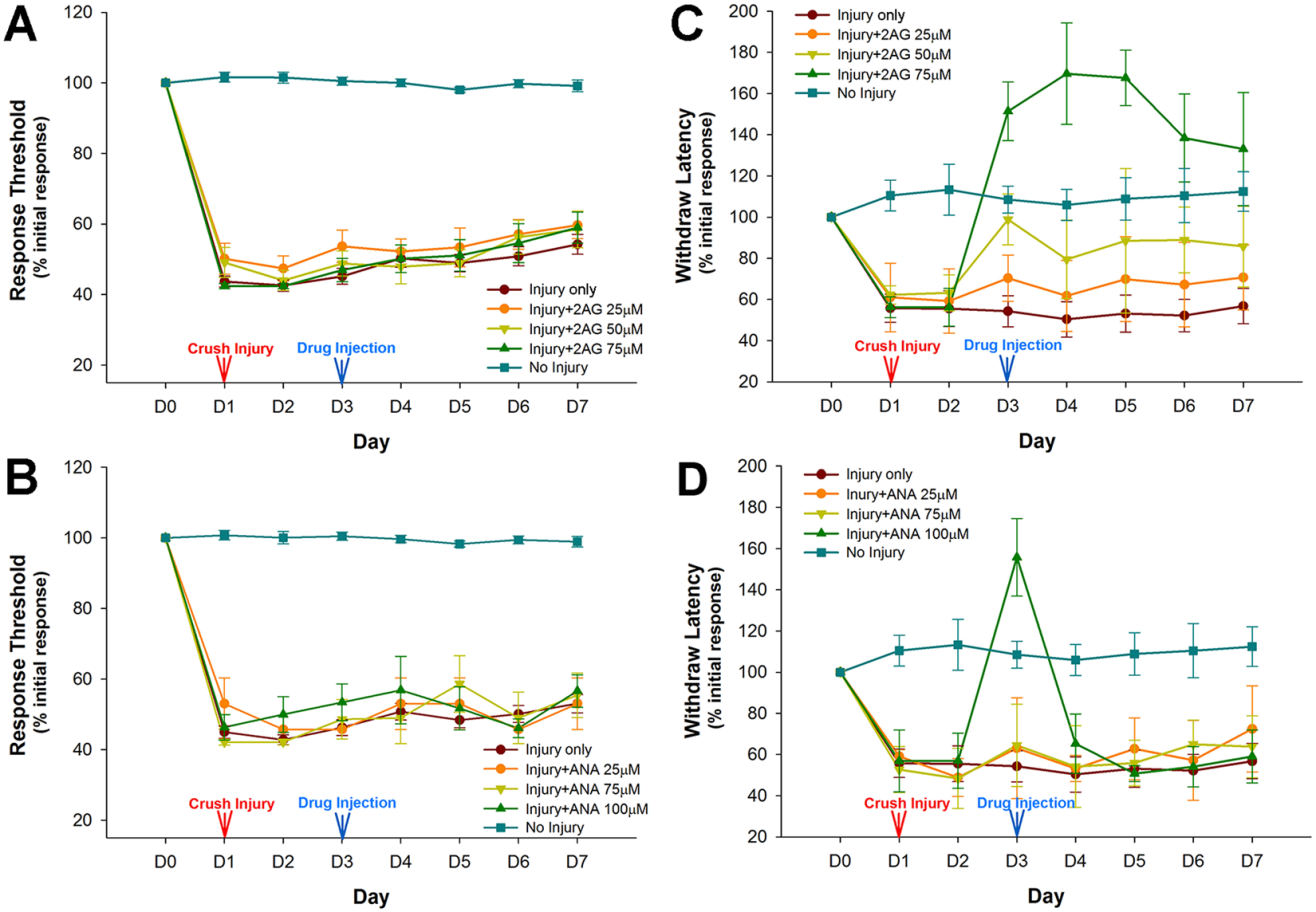
Effect of endocannabinoids on *Hirudo* that had undergone injury-induced sensitization. Hirudo were given a crush injury to the posterior sucker and tested for changes in responsiveness to nociceptive and non-nociceptive stimuli for seven days. (**A**,**B**). Crush injury to the posterior sucker sensitized these animals to non-nociceptive mechanical stimuli. Neither 2-AG (25, 50, 75 μM) nor anandamide (50, 75, 100 μM) affected responses to non-nociceptive mechanical stimuli. (**C**,**D**) This same injury sensitized animals to nociceptive chemical stimuli (AITC). This sensitization was reduced by 100 µM 2-AG and 50 µM 2-AG. 25 µM had no effect. Anandamide was also capable of reducing this injury-induced sensitization, but only at the highest concentration (75 µM).

Animals given a crush injury to the posterior sucker exhibited sensitization to non-nociceptive stimuli, expressed as a decrease in the response threshold to mechanical stimulation with the von Frey fibers. These animals also exhibited sensitization to nociceptive stimuli expressed as a reduced latency to respond to AITC application. Both types of sensitization were observed throughout the 7 day testing period (Fig. 3).

Sensitization to non-nociceptive stimuli was unaffected by 2-AG injection made on day 3 (Fig. 3A). A two-way ANOVA comparing non-injured control animals (N = 8), injured animals (N = 8) and injured animals receiving 25, 50 or 75 µM 2-AG (N = 4, 4, 7, respectively) detected a significant effect of treatment (F_4,391_ = 530.36, p < 0.001), a significant effect of time (F_6,391_ = 6.38, p < 0.001), but no significant treatment-time interaction effect (F_24,391_ = 1.39, p > 0.05). Subsequent post-hoc analysis confirmed that all of the injured groups had a significant decrease in the threshold required to elicit localized withdrawal response compared to uninjured controls (p < 0.001). However, no there was no significant difference between injury-only group and the injury + 2-AG, regardless of the 2-AG concentration (p > 0.05 for all). Anandamide treatment also failed to alter sensitization to non-nociceptive stimuli (Fig. 3B). A two-way ANOVA comparing non-injured control animals (N = 8), injured animals (N = 8) and injured animals receiving 50, 75 or 100 µM anandamide (N = 3, 3, 5 respectively) detected a significant effect of treatment (F_4,321_ = 640.30, p < 0.001), a significant effect of time (F_6,321_ = 2.62, p < 0.05), but no significant treatment-time interaction effect (F_24,321_ = 1.12, p > 0.05). Subsequent post-hoc analysis confirmed that all of the injured groups had a significant decrease in the threshold compared to the non-injured control group (p < 0.001). However, there was no significant difference between injury-only group and the injury + anandamide group, regardless of the anandamide concentration (p > 0.05 for all). Together these results indicate that 2-AG and anandamide had no effect on responses to non-nociceptive stimuli in *Hirudo* that have undergone injury-induced sensitization.

2-AG applied on day 3 did reduce sensitization to chemical nociceptive stimuli (Fig. 3C). A two-way ANOVA detected a significant effect of treatment (F_4,222_ = 35.58, p < 0.001), a significant effect of time (F_6,222_ = 3.79, p < 0.01) and a significant treatment-time interaction effect (F_24,222_ = 2.63, p < 0.01). Subsequent post-hoc analysis showed that the response latency in injured animals was significantly lower compared non-injured controls (p < 0.001). The injury + 75μM 2-AG group was significantly different from all groups including the non-injured controls (p > 0.001), until days 6 and 7 when the 2-AG-treated group was no longer statistically different from the non-injured controls (although they remained statistically different from the injured group). 50 μM 2-AG reduced sensitization to nociceptive stimuli relative to the injured control group (p < 0.01; post-hoc of treatment effect), but was also different from the non-injured control group (p < 0.05), indicating this concentration of 2-AG was not as effective as 75 μM. 25 μM 2-AG had no effect on injury-induced sensitization to nociceptive stimuli (p ≥ 0.05).

Anandamide also reduced injury-induced sensitization to nociceptive stimuli, but not as effectively as 2-AG (Fig. 3D). A two-way ANOVA detected a significant effect of treatment (F_4,193_ = 35.18, p < 0.001), a significant effect of time (F_6,193_ = 2.16, p < 0.05), but not a significant treatment-time interaction effect (F_24,193_ = 1.89, p ≥ 0.05). Subsequent post-hoc analysis showed that the response latency in injured animals was again significantly lower compared non-injured controls (p < 0.001). Only 100 μM anandamide was effective in reducing injury-induced sensitization based on a post-hoc comparison of the injury + 100 μM anandamide and injury groups (p < 0.05) and this effect was restricted to just the day of drug injection. 75 and 50 μM anandamide had no effect on injury-induced changes in response latency to nociceptive stimuli. In conclusion, while anandamide can ameliorate injury-induced sensitization, the effect is not as strong nor as long-lasting as the effect of 2-AG.

## Discussion

This study presents behavioral evidence for opposing effects of endocannabinoids in responses to non-nociceptive mechanical vs. nociceptive chemical stimuli. Specifically, 2-AG and anandamide both enhanced responses to non-nociceptive stimuli and reduced responses to nociceptive stimuli. Both of these effects were blocked when the TRPV channel inhibitor SB366791 was co-injected. We also examined the effects of endocannabinoids on animals that had undergone an injury to the posterior sucker that produced persistent (at least 7 days) sensitization to both nociceptive and non-nociceptive stimuli. 2-AG and anandamide had no effect on responses to non-nociceptive stimuli in these injury-induced sensitized animals. This lack of effect is likely due to the fact that further decreases in the response threshold cannot occur because these animals already maximally sensitized due to the injury. However, it is also possible that endocannabinoids mediated the injury-induced sensitization itself and therefore this sensitization occludes any additional effects of injected 2-AG or anandamide. High frequency stimulation of the *Hirudo* nociceptive neurons does produce endocannabinoid-mediated potentiation of the pressure cell synapses that are likely to mediate responses to the von Frey fibers used in this experiment^33^. Future studies will examine the potential role of endocannabinoid signaling in mediating injury-induced sensitization to non-nociceptive injury.

2-AG and anandamide injections increased the response latency to chemical nociceptive stimuli (AITC), an anti-nociceptive effect. Injury-induced sensitization was not observed following injection of 100 µM 2-AG and there was an increased response latency relative to non-injured control animals for several days after injection. 50 µM 2-AG produced a smaller anti-nociceptive effect, but one that still lasted several days. 25 µM 2-AG was ineffective. By comparison anandamide was less effective with only the highest concentration (100 µM) producing an anti-nociceptive effect that only lasted one day. Both 2-AG and anandamide produce similar levels of depression in *Hirudo* nociceptive synapses although this was only measured for 1–2 hrs^31, 33, 35^. It is not clear at this time why 2-AG and anandamide are so different in terms of the duration of their behavioral effects.

These results are consistent with previous *in vitro* neurophysiological studies carried out in isolated ganglia and semi-intact preparation in which endocannabinoids depress nociceptive (N cell) synapses, but potentiate non-nociceptive (P cell) synapses (see Fig. 1A)^31–33^. Pharmacological inhibitors of TRPV1 blocked both endocannabinoid-mediated potentiation of P cell synapses and depression of the N cell synapses. Previous pharmacological studies have shown that *Hirudo* possesses a TRPV-like channel both peripherally and in the CNS that responds to capsaicin and TRPV1 antagonists such as SB366791 and capsazepine^24, 31, 33^. Depression of N cell synapses is due to activation of presynaptic TRPV-like channels that is thought to lead to a decrease in neurotransmitter release (see Fig. 1A) and is calcineurin- and transcription-dependent^30, 34, 35^. These mechanisms are similar to endocannabinoid/TRPV1-mediated depression in hippocampal synapses^41, 42^. Although also TRPV-mediated, P cells lack TRPV-like channels and potentiation of P cell synapses is an indirect process that involves disinhibition of theses synapses (see Fig. 1A)^32, 33^. Nociceptive synapses are “protected” from this disinhibition because they are depolarized by GABA due to elevated levels of intracellular Cl^−^^33, 36^. At this time the GABAergic neurons in *Hirudo* that undergo this suggested endocannabinoid-mediated depression have not been identified.

An important caveat of these studies and much of the previous *Hirudo* work is that they are based on pharmacological manipulations. It is possible that the observed drug effects are due, at least in part, to off-target effects unique to invertebrates. For example, the platyhelminth *Schistosoma mansoni*, responds to capsaicin, but this effect is mediated by TRPA1 channels (which are also present in *Hirudo*)^25, 40^. We have tried to minimize this potential confound in our past studies by utilizing multiple pharmacological agents^30, 31, 33^. However, this concern will remain until *Hirudo* versions of these proteins are isolated and directly examined.

The current experiments, combined with previous synaptic studies, have relevance to understanding endocannabinoid/TRPV-based modulation of nociception. Endocannabinoids have been reported to exert an anti-nociceptive effect due at least in part to depression of glutamatergic transmission at primary afferent synapses in the spinal cord^4, 5, 7, 43, 44^. However, stimulation of CB1 receptor can also enhance nociception due to depression of GABAergic/glycinergic inhibitory transmission within the spinal cord^8^. Interestingly, injury-induced allodynia due to TRPV1-mediated disinhibition in the spinal cord has been observed although it is not known whether endocannabinoids are activating the TRPV1 channel^45^. Finally, pro-nociceptive effects are also observed in animals with a genetic knock-out of fatty acid amide hydrolase (FAAH), which is responsible for anandamide metabolism, and these effects were mediated by both CB1 and TRPV1^9^. This capacity for endocannabinoids to have both pro- and anti-nociceptive effects, potentially through both CB1- and TRPV1-mediated signaling, may help to explain why some clinical studies of cannabinoid-based therapies to treat chronic pain can sometimes result in either no effect or a worsening of symptoms^10–12^. These pro-nociceptive effects may be due in part to endocannabinoids disinhibiting (and therefore enhancing) the nociceptive circuitry. Disinhibition is a critical mechanism that “opens the gate” for non-nociceptive afferents to have access to spinal nociceptive circuits^15–17, 45^, but the mechanisms by which injury elicits disinhibition associated with sensitization of non-nociceptive pathways are not fully understood. Differences in Cl^−^ gradients between nociceptive and non-nociceptive afferents may contribute to the latter being more sensitive to disinhibition^10, 33, 36, 46–48^. In *Hirudo* this disinhibition selectively affects non-nociceptive pathways, but may have effects on both non-nociceptive and nociceptive pathways in mammals possibly due to the more complex circuitry (in terms of multiple classes of inhibitory and excitatory interneurons) at the spinal cord level^14, 15^.

Together, these findings demonstrate direct behavioral evidence for the opposing effects of endocannabinoids in both injured and non-injured animals. These results significantly contribute to understanding the potential role of endocannabinoids in both the induction and attenuation of pain conditions and demonstrate the need for more studies characterizing the specific mechanisms unique to sensitization of nociceptive sensory pathways versus sensitization of non-nociceptive pathways. From a clinical standpoint, endocannabinoid-based therapies may only have efficacy for certain types of pain conditions. Specifically, it is possible that cannabinoid-based therapies will be more effective for conditions that involve hyperalgesia, but either less effective for or perhaps even exacerbate conditions that include allodynia. Such considerations must be taken into account when designing future clinical studies that seek to use the endocannabinoid signaling system to treat chronic pain.
